# Siamese hierarchical feature fusion transformer for efficient tracking

**DOI:** 10.3389/fnbot.2022.1082346

**Published:** 2022-12-01

**Authors:** Jiahai Dai, Yunhao Fu, Songxin Wang, Yuchun Chang

**Affiliations:** ^1^Department of Electronic Information Engineering, College of Electronic Science and Engineering, Jilin University, Changchun, China; ^2^Department of Computer Science and Technology, College Science and Technology, Shanghai University of Finance and Economics, Shanghai, China; ^3^Department of Electronic Science and Technology, School of Microelectronics, Dalian University of Technology, Dalian, China

**Keywords:** visual tracking, hierarchical feature, transformer, lightweight backbone, real-time

## Abstract

Object tracking is a fundamental task in computer vision. Recent years, most of the tracking algorithms are based on deep networks. Trackers with deeper backbones are computationally expensive and can hardly meet the real-time requirements on edge platforms. Lightweight networks are widely used to tackle this issue, but the features extracted by a lightweight backbone are inadequate for discriminating the object from the background in complex scenarios, especially for small objects tracking task. In this paper, we adopted a lightweight backbone and extracted features from multiple levels. A hierarchical feature fusion transformer (HFFT) was designed to mine the interdependencies of multi-level features in a novel model—SiamHFFT. Therefore, our tracker can exploit comprehensive feature representations in an end-to-end manner, and the proposed model is capable of handling small target tracking in complex scenarios on a CPU at a rate of 29 FPS. Comprehensive experimental results on UAV123, UAV123@10fps, LaSOT, VOT2020, and GOT-10k benchmarks with multiple trackers demonstrate the effectiveness and efficiency of SiamHFFT. In particular, our SiamHFFT achieves good performance both in accuracy and speed, which has practical implications in terms of improving small object tracking performance in the real world.

## Introduction

Visual tracking is an important task in computer vision that provides underlying technical support for more complex tasks; and is an essential procedure for advanced computer vision applications. Additionally, visual tracking has been widely used in various fields such as unmanned aerial vehicles (UAVs) (Cao et al., 2021), autonomous driving (Zhang and Processing, 2021), and video surveillance (Zhang G. et al., 2021). However, several challenges remain that hamper tracking performance, including edge computing devices and difficult external environments with occlusion, illumination variation, and background clutter.

Over the past few years, visual object tracking has made significant advancements based on the development of convolutional neural networks due to the breakthroughs that have been made to generate more powerful backbones, such as deeper networks (He et al., 2016; Chen B. et al., 2022), efficient network structure (Howard et al., 2017), attention mechanism (Hu et al., 2018). Inspired by the way of the human brain process the overload information (Wolfe and Horowitz, 2004), the attention mechanism is utilized to enhance the vital features and surpass the unnecessary information of the input feature. Due to the powerful feature representation ability, the attention mechanism becomes an important means to enhance the input features, such as channel attention (Hu et al., 2018), spatial attention (Wang F. et al., 2017; Wang N. et al., 2018), temporal attention (Hou et al., 2020), global attention (Zhang et al., 2020a), and self-attention mechanism (Wang et al., 2018). Among them, the self-attention based models, the transformer was initially designed for natural language processing (NLP) (Vaswani et al., 2017) task, where the attention mechanism is utilized to perform the machine translation tasks and achieved great improvements. Later, the pre-training model BERT (Devlin et al., 2018) achieve breakthrough progress in NLP tasks, further advance the development of the Transformer model. Since then, both academia and industry have set off a boom in the research and application of pre-trained models based on Transformer, and gradually extended from NLP to CV. For example, Vision Transformer (ViT) (Dosovitskiy et al., 2020), DETR (Carion et al., 2020), have surpassed previous SOTA in the fields of image classification, inspection, and video, respectively. Various variant models based on Transformer structure have been proposed, multi-task indicators in various fields have been continuously refreshed, and the deep learning community has entered a new era. Meanwhile, muti-level features fusion can effectively alleviate the deficiency of the transformer in handling the tracking of small objects.

Although transformer models provide enhancements in feature representation and result in promotion in terms of accuracy and robustness, trackers based on transformers have high computational costs that hinder them from meeting the real-time demands of tracking tasks on edge hardware devices, providing a disadvantage for the landing of the application. Therefore, how to balance the efficiency and efficacy of object trackers remains a significant challenge. Generally, discriminative feature representation is essential for tracking. Therefore, deeper backbones and online updaters are utilized in tracking frameworks, however these methods are computationally expensive leading to increased run time and budget. Typically, the lightweight backbone is also limited as it typically provides inadequate feature extraction, rendering the tracking model less robust for small objects or complex scenarios.

In this study, we employed a lightweight backbone network to avoid the efficiency loss caused by the computations of deep networks. To address the insufficient feature representations extracted by shallow networks, we extracted features from multiple levels of the backbone to enrich the feature representations. Furthermore, to leverage the advantages of transformers in global relationship modeling, we designed a hierarchical feature fusion module to integrate multi-level features comprehensively using multi-head attention mechanisms. The proposed Siamese hierarchical feature fusion transformer (SiamHFFT) tracker achieved robust performance in complex scenarios while maintaining real-time tracking speed on a CPU and it can be deployed on consumer CPUs. The main contributions of this study can be summarized as follows:

(1) We proposed a novel type of tracking network based on a Siamese architecture, which consisting of feature extraction, reshape module, Transformer-like feature fusion module, and head prediction modules.
(2) We designed a feature fusion transformer to exploit the hierarchical features in the Siamese tracking framework in an end-to-end manner, which is capable of advancing discriminability for small object tracking task.
(3) Comprehensive evaluations on five challenging benchmarks demonstrate the proposed tracker achieved promising results among state-of-the-art trackers. Besides, our tracker can run at a real-time speed. This efficient method can be deployed on resource-limited platforms.

The remainder of this paper is organized as follows. Section Related work describes related work on tracking networks and transformers. Section Method introduces the methodology used for implementing the proposed HFFT and network model. Section Experiments presents the results of experiments conducted to verify the proposed model. Finally, Section Conclusion contains our concluding remarks.

## Related work

### Siamese tracking

In recent years, Siamese-based networks have become a ubiquitous framework in the visual tracking field (Javed et al., 2021). Tracking an arbitrary object can be considered as learning similarity measure function learning problems. SiamFC (Bertinetto et al., 2016) introduced a correlation layer as a fusion tensor into the tracking framework for the first time, which pioneered the Siamese tracking procedure. Instead of directly estimating the target position according to the response map, SiamRPN (Li B. et al., 2018) attaches a region proposal extraction subnetwork (RPN) to the Siamese network and formulates the tracking as a one-shot detection task. Based on the results of classification and regression branches, SiamRPN achieves enhanced tracking accuracy. DaSiamRPN (Zhu et al., 2018) uses a distractor-aware module to solve the problem of inaccurate tracking caused by the imbalance of positive and negative samples of the training set. C-RPN (Fan and Ling, 2019) and Cract (Fan and Ling, 2020) incorporate multiple stages into the Siamese tracking architecture to improve tracking accuracy. To address unreliable predicted fixed-ratio bounding boxes when a tracker drifts rapidly, an anchor-free mechanism was also introduced into the tracking task. To rectify the inaccurate bounding box estimation strategy of the anchor-based mechanism, Ocean (Zhang et al., 2020b) directly regresses the location of each point located in the ground truth. SiamBAN (Chen et al., 2020) adopts box adaptive heads to handle the classification and regression problem parallelly. SiamFC++ (Xu et al., 2020) and SiamCAR (Guo et al., 2020) draw on the FCOS architecture and add a branch to measure the accuracy of the classification results. Compared with anchor-based trackers, anchor-free-based trackers utilize fewer parameters and do not need prior information for the bounding box, these anchor-free-based trackers can achieve a real-time speed.

As feature representation plays a vital role in the tracking process (Marvasti-Zadeh et al., 2021), several works delicate to obtain discriminative features from different perspectives, such as adopting deeper or wider backbones, and using attention mechanisms to advance the feature representation. In the recent 3 years, the Transformer is capable of using global context information and preserving more semantic information. The introduction of the Transformer model in the tracking community boots the tracking accuracy to a great extent (Chen X. et al., 2021; Lin et al., 2021; Liu et al., 2021; Chen et al., 2022b; Mayer et al., 2022). However, the promotion of the accuracy of these trackers' increasingly complex models relies heavily on powerful GPUs, leading to the inability to deploy such models on edge devices, which hinders the further practical application of the models.

In this study, to optimize the trade-off between tracking accuracy and speed, we designed an efficient algorithm that employs a concise model consisting of a lightweight backbone network, a feature reshaping model, a feature fusion module, and a prediction head. Our model is capable of handling complex scenarios, and the proposed tracker can also achieve real-time speed on a CPU.

### Transformer in vision tasks

As a new type of neural network, transformer shows superior performance in the field of AI applications (Han et al., 2022). Unlike the structure of CNNs and RNNs, Transformer adopts the self-attention mechanism, which has been proved to have strong feature representation ability and better parallel computing capability, making it more advantageous in several tasks.

The transformer model was first proposed by Vaswani et al. (2017) for application to natural language processing (NLP) tasks. In contrast to convolutional neural networks (CNNs) and recurrent neural networks (RNNs), self-attention facilitates both parallel computation and short maximum path lengths. Unlike earlier self-attention models based on RNNs for input representations (Lin Z. et al., 2017; Paulus et al., 2017), the attention mechanisms in transformer model are implemented with attention-based encoders and decoders instead of convolutional or recurrent layers.

Because transformers were originally designed for sequence-to-sequence learning on textual data and have exhibited good performance, their ability to integrate global information has been gradually unveiled and transformers have been extended to other modern deep learning applications such as image classification (Liu et al., 2020; Chen C. -F. R. et al., 2021; He et al., 2021), reinforcement learning (Parisotto et al., 2020; Chen L. et al., 2021), face alignment (Ning et al., 2020), object detection (Beal et al., 2020; Carion et al., 2020), image recognition (Dosovitskiy et al., 2020) and object tracking (Yan et al., 2019, 2021a; Cao et al., 2021; Lin et al., 2021; Zhang J. et al., 2021; Chen B. et al., 2022; Chen et al., 2022b; Mayer et al., 2022). Based on CNNs and transformers, the DERT (Carion et al., 2020) applies a transformer to object detection tasks. To improve upon previous CNN models, DERT eliminates post-processing steps that rely on manual priors such as non-maximum suppression (NMS) and anchor generators; and constructs a complete end-to-end detection framework. ViT (Dosovitskiy et al., 2020) mainly converts images into serialized data through token processing and introduces the concept of patches, where input images are divided into smaller patches and each patch is converted into a bidirectional encoder representation from transformers-like structure. Similar to the concept of patches in ViT, Swin Transformer (Liu et al., 2021) uses the concept of windows, but the calculations of different windows do not interfere with each other, hence, the computational complexity of the Swin Transformer is significantly reduced.

In the tracking community, transformers have achieved remarkable performance. STARK (Yan et al., 2021a) utilizes an end-to-end transformer tracking architecture based on spatiotemporal information. SwinTrack (Lin et al., 2021) incorporates a general position-encoding solution for feature extraction and feature fusion, enabling full interaction between the target object and search region during tracking process. TrTr (Zhao et al., 2021) used the transformer architecture to perform target classification and bounding box regression and designed a plug-in online update module for classification to further improve tracking performance. DTT (Yu et al., 2021) also feed these architectures to predict the location and the bounding box of the target. Cao et al. (2021) proposed an efficient and effective hierarchical feature transformer (HiFT) for aerial tracking. HCAT (Chen et al., 2022b) utilizes a novel feature sparsification module to reduce computational complexity and a hierarchical cross-attention transformer that employs a full cross-attention structure to improve efficiency and enhance representation ability. The hierarchical-based methods, both HiFT and HCAT show good tracking performance. However, transformer-based trackers lack robustness in small objects. In this paper, we propose a novel hierarchical feature fusion module based on a transformer to enable a tracker to achieve real-time speed while maintains good accuracy.

### Feature aggregation network

Feature aggregation plays a vital role in the multi-level feature process, and is used to improve cross-scale feature interaction and multi-scale feature fusion, thereby enhancing the representation of features and enhancing network performance. Zhang G. et al. (2021) proposed a hierarchical aggregation transformer (HAT) framework consisting of transformer-based feature calibration (TFC) and deeply supervised aggregation (DSA) modules. The TFC module can merge and preserve semantic and detail information at multiple levels, and the DSA module aggregates the hierarchical features of the backbone with multi-granularity supervision. Feature pyramid networks (FPN) (Lin T.-Y. et al., 2017) introduce cross-scale feature interactions and achieve good results through the fusion of multiple layers. Qingyun et al. (2021) introduced a cross-modality fusion transformer, that makes full use of the complementarity between different modalities to improve the performance of features. However, the main challenge of a simple feature fusion strategy is how to fuse high-level semantic information and low-level detailed features. To address these issues, we propose an aggregation structure based on hierarchical transformers, which can fully mine the coherence among multi-level features at different scales, and achieve discriminative feature representation ability.

## Method

### Overview

**Figure 1:**
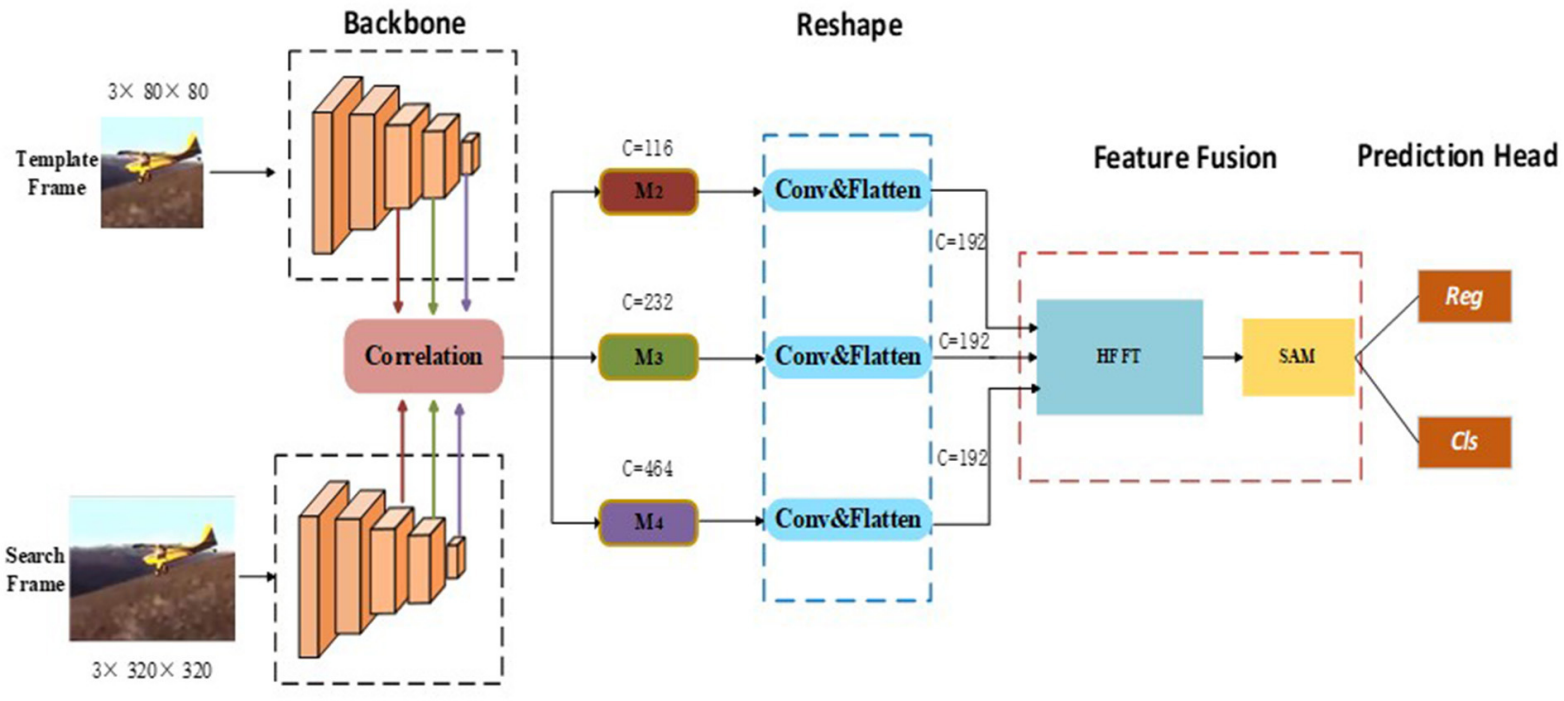
Architecture of the proposed SiamHFFT tracking framework. This framework contains four fundamental components: a feature extraction network, reshaping module, feature fusion module, and prediction head. The backbone network is used to extract hierarchical features. The reshaping module is designed to perform convolution operations and flatten features. The feature fusion transformer consists of the proposed HFFT module and a self-attention module (SAM). Finally, bounding boxes are estimated based on the regression and classification results.

In this section, we describe the proposed SiamHFFT model. As can be seen in Figure 1, our model follows a Siamese tracking framework. There are four key components in our model, namely the feature extraction module, reshape module, feature fusion module, and prediction head. During tracking, the feature extraction module extracts feature from the template and search region. The features of the two branches from the last three layers of the backbone are correlated separately, and the outputs are denoted as *M*_2_, *M*_3_, and *M*_4_ in order. We then feed the correlated features into the reshaping module, which can transform the channel dimensions of the backbone features and flatten features in the spatial dimension. The feature fusion module is implemented by fusing features using our hierarchical feature fusion transformer (HFFT) and a self-attention module. Finally, we used the prediction head module to perform bounding box regression and binary classification on the enhanced features to generate tracking results.

### Feature extraction and reshaping

Similar to most Siamese tracking networks, the proposed method uses template frame patch (*Z* ∈ ℝ^3×80×80^) and search frame patch (*X* ∈ ℝ^3×320×320^) as inputs. For the backbone, our method can use an arbitrary deep CNN such as ResNet, MobileNet (Sandler et al., 2018), AlexNet, or ShuffleNet V2 (Ma et al., 2018). In this study, because a deeper network is unsuitable for deployment with limited computing resources, we adopted ShuffleNetV2 as a backbone network. This network is utilized for both template and search branch feature extraction.

To obtain robust and discriminative feature representations, we incorporate detailed structural information into our visual representations by extracting hierarchical features with different scales and semantic information in stage two, three and four of feature extraction. We denote feature tokens from the template branch as *F*_*i*_(*Z*) and those from the search branch as *F*_*i*_(*X*), where *i* represents the stage number of feature extraction and *i* ∈ {2, 3, 4}.

Next, a convolution operation is performed on the feature maps from the multi stages correlation, which is defined as:


(1) $$\begin{array}{l} M_{i}=F_{i}\left(Z\right)*F_{i}\left(X\right),i=2,3,4, \end{array}$$

where $M_{i}\in {\mathbb{R}}^{C_{i}\times H_{i}\times W_{i}}$, and *C*, *H*, and *W* denote the channel, width, and height of the feature map respectively. Additionally, *C*_*i*_ ∈ {116, 232, 464} and * denotes the cross-correlation operator. Next, we use the reshaping module which consists of 1 × 1 convolutional kernels, to change the channel dimensions of the features from Equation (1). We then flatten the features in the spatial dimension because a unified channel can not only effectively reduce computing resource requirements, but is also an essential component for improving the performance of feature fusion. After these operations, we can obtain a reshaped feature map ${M_{i}}^{\prime }\in {\mathbb{R}}^{W_{i}H_{i}\times C}$, where *C* = 192.

### Feature fusion and prediction head

As illustrated in Figure 1, following the convolution and flattening operations in the reshaping module, the correlation features from different stages are unified in the channel dimension. To explore the interdependencies among multi-level features fully, we designed the HFFT, which is detailed in this section.

**Figure 2:**
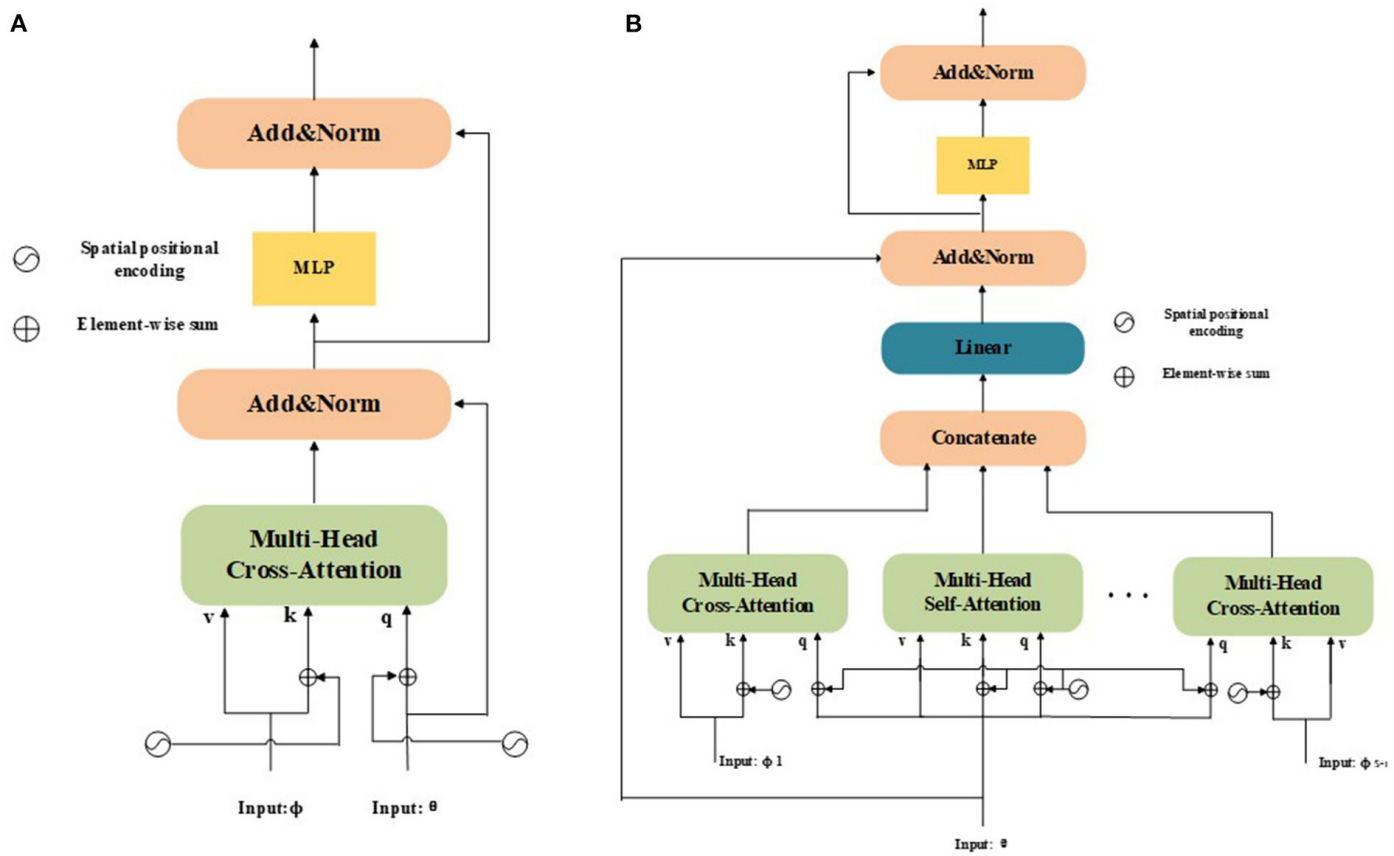
**(A)** Structure of a dual-input tasks; **(B)** Structure of a multi-input tasks. Unlike the original dual-input tasks, multi-input tasks can be used to learn the interdependencies of multi-level features and enhance the feature representation of the model in an end-to-end manner.

**Multi-Head Attention (Vaswani et al.**, **2017****):** Generally, transformers have been successfully applied to enhance feature representations in various bi-modal vision tasks. In the proposed feature fusion module, the attention mechanism is also a fundamental component. It is implemented using an attention function and operated on queries *Q*, keys *K* and values *V* using the scale dot-production method, which is defined as:


(2) $$\begin{array}{l} Attention\left(Q,K,V\right)=softmax\left(\frac{QK^{⊤}}{\sqrt{C}}\right)V \end{array}$$

where *C* is the key dimensionality for normalizing the attention, and$\sqrt{C}$ is a scaling factor to avoid gradient vanishing in the loss function. Specifically, $Q={\left[q_{1},\ldots ,q_{N}\right]}^{T}\in {\mathbb{R}}^{N\times C}$ is the *q* input in Figure 2B, which denotes a collection of *N* features; similarly, *K* and *V* are the *k* and *v* inputs, respectively, which represent a collection of *M*features (i.e., *K, V* ∈ ℝ^*M*×*C*^). Notably, *Q, K, V* represent the mathematical implementation of the attention function and do not have practical meaning.

According to Vaswani et al. (2017), extending the attention function in Equation (2) to multiple heads is beneficial for enabling the mechanism to learn various attention distributions and enhancing its feature representation ability. This extension can be formulated as follows:


(3) $$\begin{array}{l} MultiHead\left(Q,K,V\right)=Concat\left(head_{1},\ldots head_{h}\right)W^{o} \end{array}$$

(4) $$\begin{array}{l} head_{i}=Attention\left(QW_{i}^{Q},KW_{i}^{K},VW_{i}^{V}\right),i=1,\ldots h \end{array}$$

where $W_{i}^{Q}$, $W_{i}^{K}$ and $W_{i}^{V}\in {\mathbb{R}}^{C\times d_{h}}$, and *W*^*o*^ ∈ ℝ^*C*×*C*^. Here, *h* is the number of attention heads, which is defined as $d_{h}=\frac{C}{h}$. In this study, we adopted and *h* = 6 as default values.

**Application to Dual-Input Tasks:** The structure of a dual-input task is presented in Figure 2A, where *Q, K*, and *V* for normal NLP/vision tasks (Nguyen et al., 2020) share the same modality. In recent years, this mechanism has been extended to dual-inputs and applied to vision tasks (Chen X. et al., 2021; Chen et al., 2022a,b). However, the original attention mechanism cannot distinguish between the position information of different input feature sequences. The original mechanism only considers the absolute position and adds absolute positional encodings to inputs. It considers the attention from a source feature ϕ to a target feature θ as:


(5) $$\begin{array}{l} A_{\phi }\left(\theta \right)=MultiHead\left(\theta +P_{\theta },\phi +P_{\phi },\phi \right) \end{array}$$

where *P*_θ_ and *P*_ϕ_ are the spatial positional encodings of features θ and ϕ, respectively. Spatial positional encoding is generated using a sine function. Equation (5) can be used not only as a single-direction attention enhancement, but also as a co-attention mechanism in which both directions are considered. Furthermore, self-attention from a feature to itself is also defined as a special case:


(6) $$\begin{array}{l} A_{\theta }\left(\theta \right)=MultiHead\left(\theta +P_{\theta },\theta +P_{\theta },\theta \right) \end{array}$$

As shown in Figure 2A, following Equations (5) and (6), the designed transformer blocks are processed independently. Therefore, the two modules can be used sequentially or in parallel. Additionally, a multilayer perceptron (MLP) module is used to enhance the fitting ability of the model. The MLP module is a fully connected network consisting of two linear projections with a Gaussian error linear unit (GELU) activation function between them, which can be denoted as:


(7) $$\begin{array}{l} MLP\left(\theta^{\prime }\right)=FC_{2}\left(GELU\left(FC_{1}\left(\theta^{\prime }\right)\right)\right) \end{array}$$

**Application to Multi-Input Tasks**: To extend the attention mechanism to multiple inputs that are capable of handling multimodal vision tasks, pyramid structures, etc., we denote the total input number as S. The structure of a multi-input task is presented in Figure 2B. If we consider each possibility, there are a total of *S*(*S* − 1) source-target cases and *S* self-attention cases. Now, we denote the multiple inputs as {θ, ϕ_1_, …, ϕ_*S*−1_}, where the target θ ∈ ℝ^*N*×*C*^ and source $\phi_{i}\in {\mathbb{R}}^{M\times C}$. Notably, θ and ϕ_*i*_ must have the same size as *C*. We then compute all the source-target cases as {*A*_ϕ_1__(θ), …, *A*_ϕ_*S*−1__(θ)}. Next, we concatenate all source-to-target attention cases with self-attention *A*_θ_(θ), which can be formulated as:


(8) $$\begin{array}{l} \theta_{concat}=\left[A_{\theta }\left(\theta \right),A_{\phi_{1}}\left(\theta \right),\ldots ,A_{\phi_{S-1}}\left(\theta \right)\right] \end{array}$$

where $\theta_{concat}\in {\mathbb{R}}^{N\times SC}$. After concatenation, the dimensions of the enhanced features in the channel change to match the size *SC* of the original feature. To accelerate these calculations further, we apply a fully connected layer to reduce the channel dimensions to:


(9) $$\begin{array}{l} {\theta_{concat}}^{\prime }=Linear\left[\theta_{concat}\right] \end{array}$$

where ${\theta_{concat}}^{\prime }\in {\mathbb{R}}^{N\times C}$. Through this process, we can obtain more discriminative features efficiently by aggregating features from different attention mechanisms.

**HFFT**: As is shown in Figure 2B, in our model, we make full use of the hierarchical features ${M_{i}}^{\prime }\in {\mathbb{R}}^{W_{i}H_{i}\times C}$ (*i* ∈ {2, 3, 4}) and generate tracking-tailored features. To integrate low-level spatial information with high-level semantic information, we feed the reshaped features from the output of Equation (1), namely ${M_{2}}^{\prime }$, ${M_{3}}^{\prime }$, and ${M_{4}}^{\prime }$, into the HFFT module, where ${M_{3}}^{\prime }$ is used for target feature, ${M_{2}}^{\prime }$ and ${M_{4}}^{\prime }$ represent source features. The importance of different aspects feature information is assigned by applying the cross-attention operator to ${M_{2}}^{\prime }$ and ${M_{4}}^{\prime }$, which is beneficial for obtaining more discriminative features. We apply self-attention to ${M_{3}}^{\prime }$, which can preserve the details of target information during tracking. Furthermore, positional information is encoded during the calculation process to enhance spatial information during the tracking process. The attention mechanisms are implemented using the operation of *K, Q, V*. Then, comprehensive features can be obtained by concatenating the outputs. Due to the complexity of a model increases with its input size, a fully connected layer is utilized to resize outputs. We also adopt residual connections around each sub-layer. Additionally, we use an MLP module to enhance the fitting ability of the model, and layer normalization (LN) is performed before the MLP and final output steps. The entire process of the HFFT can be expressed as:


$$\begin{array}{l} M_{concat}=\left[A_{{M_{3}}^{\prime }}\left({M_{3}}^{\prime }\right),A_{{M_{2}}^{\prime }}\left({M_{3}}^{\prime }\right),A_{{M_{4}}^{\prime }}\left({M_{3}}^{\prime }\right)\right], \end{array}$$

$$\begin{array}{l} {M_{concat}}^{\prime }=Linear\left[M_{concat}\right], \end{array}$$

$$\begin{array}{l} M_{out}=LN\left({M_{concat}}^{\prime }+{M_{3}}^{\prime }\right), \end{array}$$

(10) $$\begin{array}{l} X_{out}=LN\left(M_{out}+MLP\left(M_{out}\right)\right) \end{array}$$

**Figure 3:**
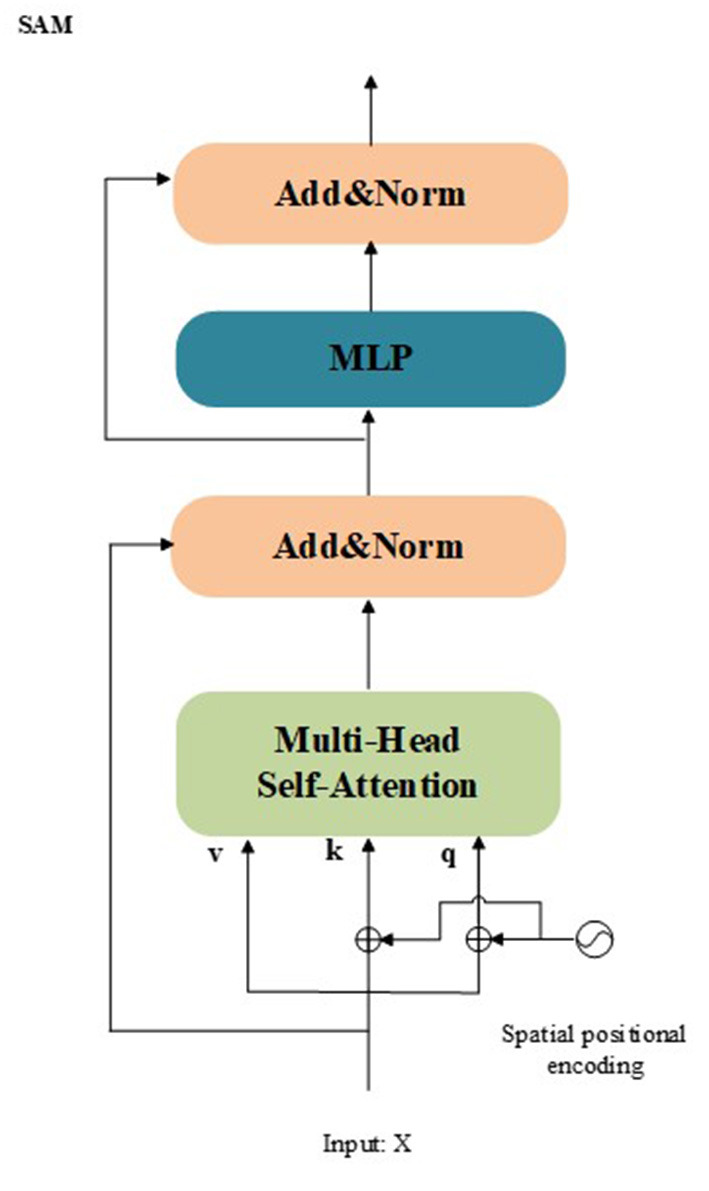
Architecture of the proposed SAM.

**SAM**: The SAM is a feature enhancement module. The structure of the SAM is presented in Figure 3. The SAM adaptively integrates information from different feature maps using multi-head self-attention in the residual form. In the proposed model, the SAM take the out of Equation (10) *X*_*out*_ as input. The mathematical process of the SAM can be summarized as:


$$\begin{array}{l} X_{out2}=LN\left(MultiHead\left(X_{out}+P_{X},X_{out}+P_{X},X_{out}\right)+X_{out}\right), \end{array}$$

(11) $$\begin{array}{l} X_{SAM}=LN\left(MLP\left(X_{out2}\right)+X_{out2}\right) \end{array}$$

**Prediction Head**: The enhanced features are reshaped back to the original feature size before being fed into the prediction head. The head network consists of two branches: a classification branch and bounding box regression branch. Each branch consists of a three-layer perceptron. The former is utilized to distinguish the target from the background, and the latter is used for estimating the location of the target by using a bounding box. Overall, the model is trained using a combination loss function formulated as:


(12) $$\begin{array}{l} L=\lambda_{cls}L_{cls}+\lambda_{giou}L_{giou}+\lambda_{loc}L_{loc} \end{array}$$

where *L*_*cls*_, *L*_*giou*_, and *L*_*loc*_ represent the binary cross-entropy, GIOU loss, and L1-norm loss, respectively. λ_*cls*_, λ_*giou*_, and λ_*loc*_ are coefficients that balance the contributions of each type of losses.

## Experiments

This section presents the details of the experimental implementation of the proposed model. To validate the performance of the proposed tracker, we compared our method to state-of-the-art methods on four popular benchmarks. Additionally, ablation studies were conducted to analyse the effectiveness of key modules.

### Implementation details

The tracking algorithm was implemented in Python based on PyTorch. The proposed model was trained on a PC with an Intel i7-11700k, 3.6 GHz CPU, 64 GB of RAM, and an NVIDIA 3080Ti RTX GPUs. The training splits of LaSOT (Fan et al., 2019), GOT-10k (Huang et al., 2019), COCO (Lin et al., 2014), and TrackingNet (Muller et al., 2018) were used to train the model. We randomly selected two image pairs from the same video sequences with a maximum gap of 100 frames to generate the search patches and template patches. The sizes of search patches were set to 320 × 320 × 3 and template patches were resized to sizes of 80 × 80 × 3. The parameters for the backbone network were initialized using ShuffleNetV2, which was pretrained on ImageNet. All models were trained for 150 epochs with a batch size of 32. Each epoch contained 60,000 sampling pairs. The coefficient parameters in Equation (12) were set to λ_*cls*_ = 2, λ_*giou*_ = 2, and λ_*loc*_ = 5. In the offline training phrase, the parameters of the model are optimized by ADAMW optimizer. The learning rates of the backbone network were set to le-5, and le-4 for the remaining parts.

### Comparisions to state-of-the-art methods

We compared SiamHFFT to state-of-the-art trackers on four benchmarks: LaSOT, UAV123 (Mueller et al., 2016), UAV123@10fps, and VOT2020 (Kristan et al., 2020). The evaluation results are presented in the following paragraphs. It is worthy note that the performance (accuracy and success scores) of the comparision methods on these compared benchmarks are obtained by the public tracking results files, which are released by their authors.

**Evaluation on LaSOT:** LaSOT is a large-scale long-term tracking benchmark consisting of 1,400 sequences. We used test splits and the one pass evaluation (OPE) to evaluate the performances of the compared trackers. That is, initialize the tracking algorithm according to the target position given in the first frame of the video sequence, and then run the prediction of the target position and size in the whole video to obtain the tracking accuracy or success rate.

**Figure 4:**
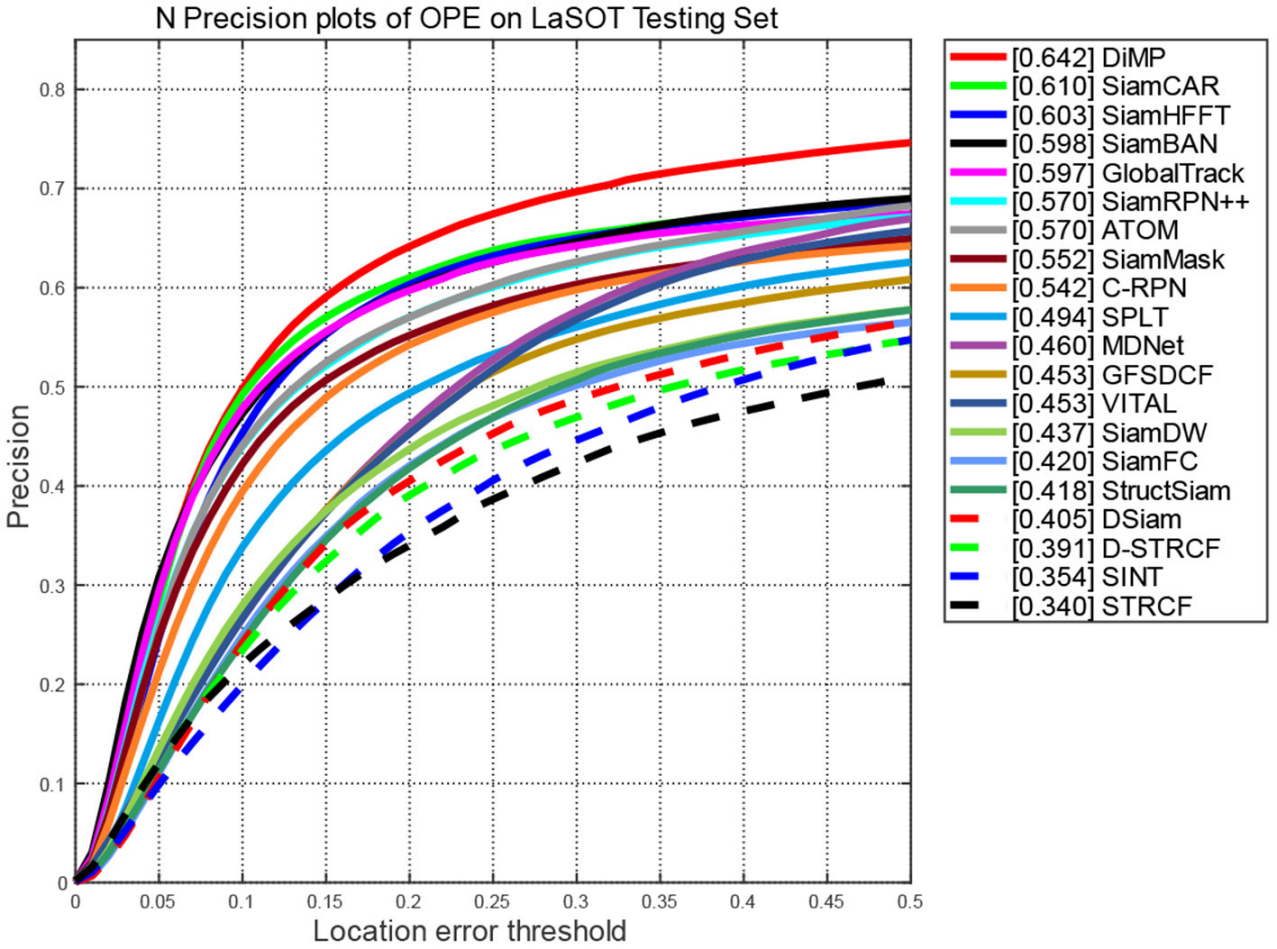
Precision scores of compared trackers on LaSOT.

**Figure 5:**
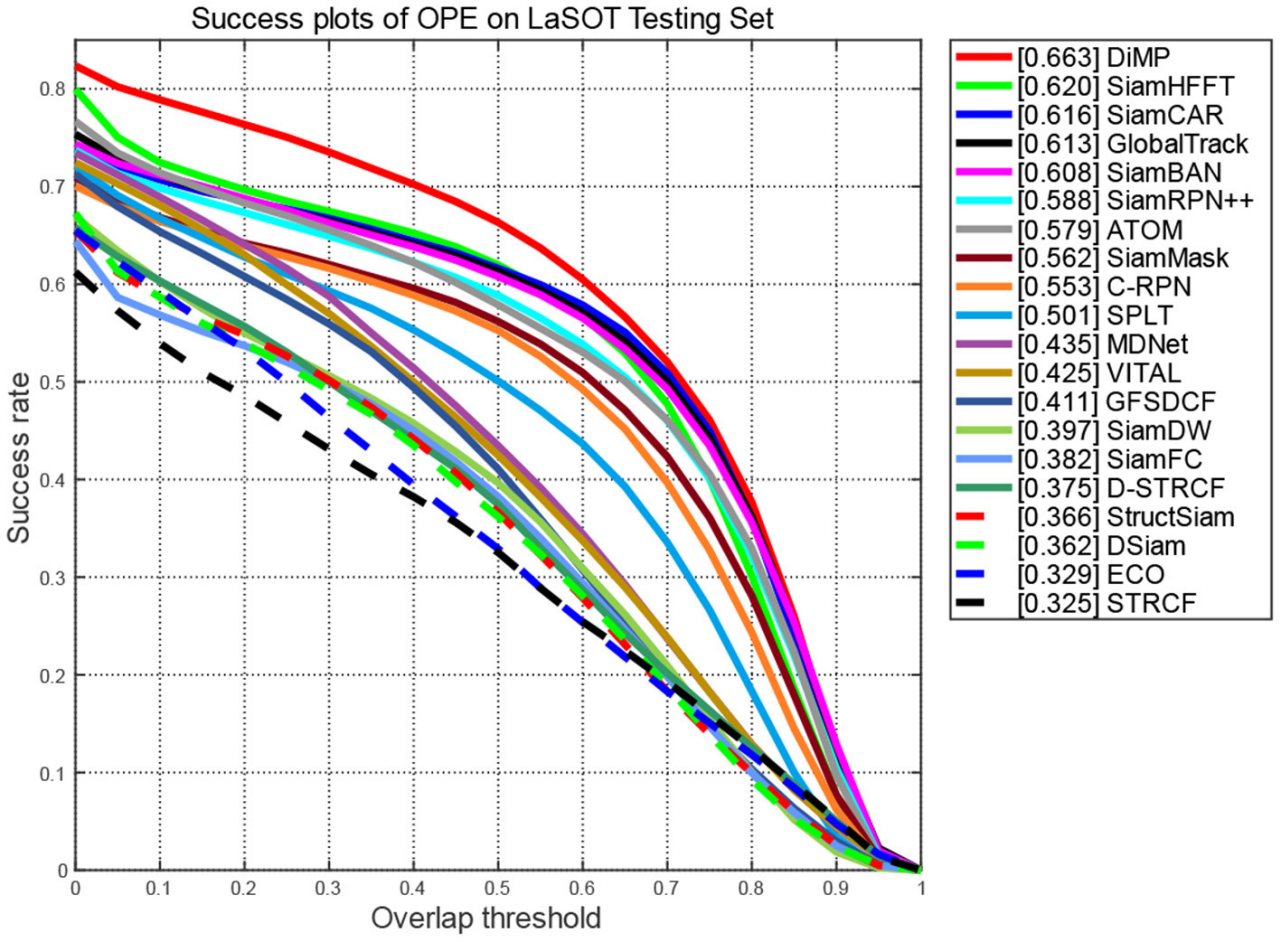
Success scores of compared trackers on LaSOT.

Figures 4, 5 report the plots of the precision and success scores of the comparision trackers, respectively. The precision score measures the center location error (CLE), which calculates the average Euclidean distance between the estimated bounding box and the ground truth. The CLE is calculated as follows:


(13) $$\begin{array}{l} CLE\;\text{=}\;\sqrt{{\left(x_{a}-x_{b}\right)}^{2}+{\left(x_{a}-x_{b}\right)}^{2}} \end{array}$$

As the CLEs of frame are obtained, the precision plots (Figure 4) show the percentage of frames in which the estimated CLE is lower than a certain threshold (usually set to 20 pixels) in the total frames of the video sequence.

The Success curve (Figure 5) refers to the percentage of the number of frames whose predicted overlap rate between the estimated bounding box and the ground truth is higher than the given threshold (usually set to 0.5) to the total number of frames in the video sequence. The overlap rate is calculated as follows:


(14) $$\begin{array}{l} S=\frac{\left|b_{t}\cap b_{g}\right|}{\left|b_{t}\cup b_{t}\right|} \end{array}$$

where *b*_*t*_ denotes the estimated bounding box, *b*_*g*_ represents the ground truth bounding box, ∩ refers to intersection operator, ∪ stands for union operator, and || denotes the number of pixels in the resulted region.

The curves of the proposed SiamHFFT are depicted in green. Overall, our tracker ranks the third in precision, and achieves the second-best score in success, with 61% at the precision score and 62% success score. Compared with the trackers with deeper backbones, such as SiamCAR, SiamBAN, and SiamRPN++ (Li B. et al., 2019), our tracker exhibits competitive performance with a lighter structure. The DiMP achieves the best performance both in precision and success. Our SiamHFFT tracker outperforms other Siamese-based trackers, even with deeper backbones and dedicated-designed structures.

**Evaluation on UAV123:** UAV123 is an aerial tracking benchmark consisting of 123 videos containing small objects, target occlusions, out of view, and distractors. To validate the performance of our tracker, we evaluated the performances of our trackers and other state-of-the-art trackers, including SiamFC, ECO (Danelljan et al., 2017), ATOM (Danelljan et al., 2019), SiamAttn (Yu et al., 2020), SiamRPN++, SiamCAR, DiMP (Bhat et al., 2019), SiamBAN, and HiFT. Table 1 lists the results in terms of success, precision, and speed on GPU. Additionally, the backbones of the trackers are also reported for an intuitive comparision. The best performance for each criterion is indicated in red.

**Table 1:** Quantitative evaluation on UAV123 in term of precision (Prec.), success (Succ.) and GPU speed (FPS).

	**SiamFC**	**ECO**	**ATOM**	**SiamAttn**	**SiamRPN++**	**SiamCAR**	**DiMP**	**SiamBAN**	**HiFT**	**SiamHFFT**
Feat.	Alex	VGG	R18	R50	R50	R50	R50	R50	Alex	ShuffleNet
Prec.	72.5	75.2	83.7	84.5	76.9	76	84.9	83.3	78.7	82.9
Succ.	49.4	52.8	64.2	65	57.9	61.4	65.4	63.1	58.9	62.6
FPS	130	45	46	45	35	52	45	40	/	68

The best performance are shown in red.

Among the trackers, those with deeper backbones, such as DiMP, ATOM, and SiamBAN, achieve better performance in term of both precision and success rate. SiamFC, HiFT, and the proposed SiamHFFT utilize lightweight backbone. SiamFC achieves the best performance in speed, but this naive network structure does not achieve satisfactory results in terms of precision and success rate. HiFT adopts a feature transformer to enhance feature representations. Compared to HiFT, our tracker exhibits a clear advantage in term of precision (82.8 vs. 78.7%) and success rate (62.5 vs. 58.9%), which validates the effectiveness of the proposed tracker. According to the last row in Table 1, all compared trackers can run in real-time on a GPU at an average speed of 68 FPS, proving that SiamHFFT maintains a suitable balance between performance and efficiency.

**Figure 6:**
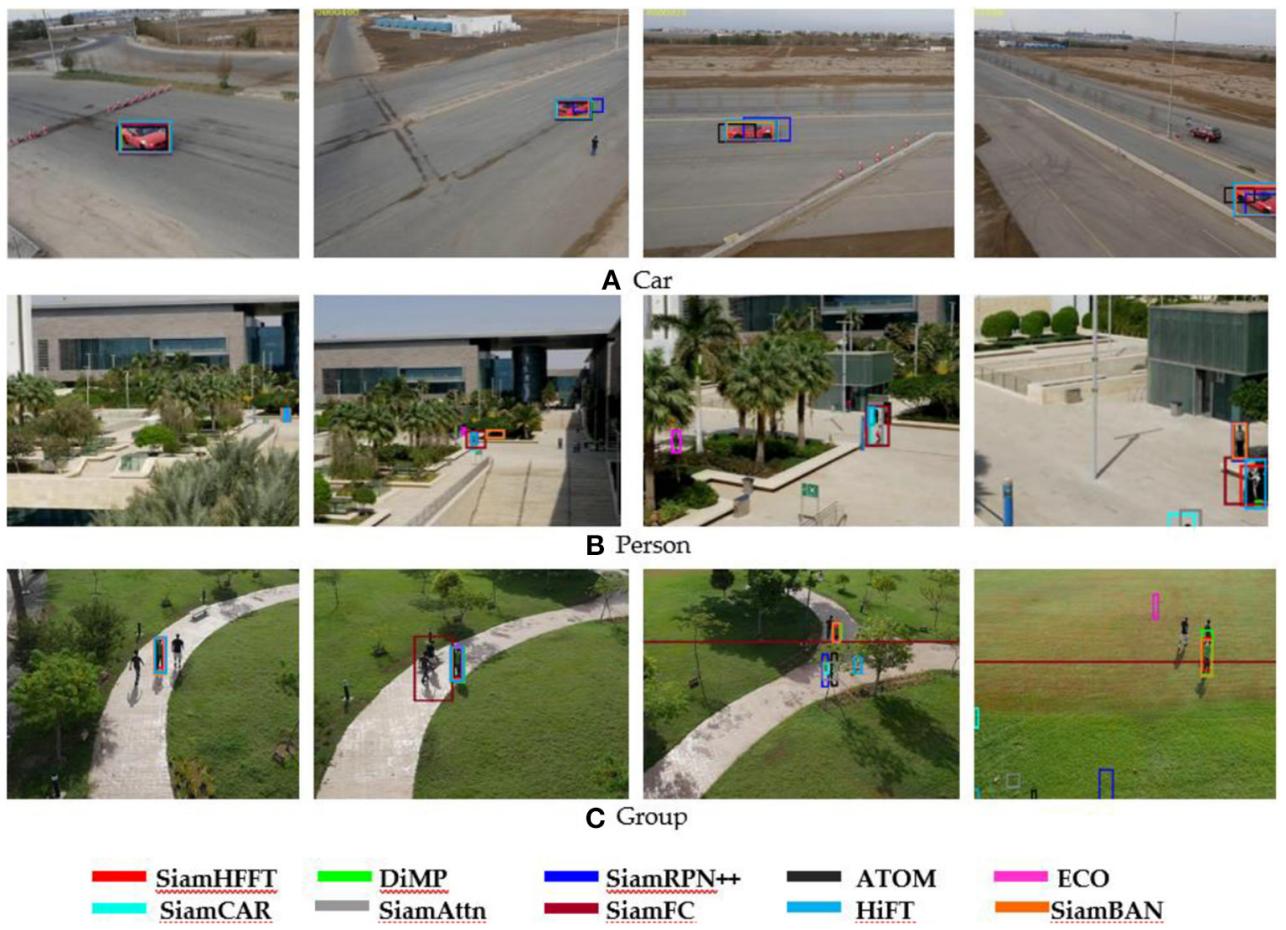
Qualitative experimental results in several challenging sequences on UAV123 dataset. **(A)** Video sequences of the Car, **(B)** video sequences of the Person, and **(C)** video sequences of the Group.

Figure 6 depicts the qualitative results by multiple algorithms on a subset of sequences in UAV123 benchmarks. We choose three sets of the challenging video sequences: Car18_1, Person21_1, and Group3_4_1. All of the three video sequences are shot by the camera of the UAV, the video frames undergo multiple challenges, for example scale variation, changes of different viewpoint, and so on. Generally, the given target appears in small size during the tracking process. The bounding boxes estimated by the trackers are marked in different colors to give an intuitive contrast. The bounding box of our SiamHFFT is shown in red, and it is obvious that our tracker can handle these complex scenarios well, especially for the small object tracking task.

**UAV123@10fps**: UAV123@10fps is a subset of UAV123 obtained by down-sampling the original videos with an image rate of 10 FPS. We use SiamFC, AutoTrack (Li et al., 2020), TADT (Li X. et al., 2019), MCCT (Wang et al., 2018), SiamRPN++, DeepSTRCF (Li F. et al., 2018), CCOT (Danelljan et al., 2016), ECO, and HIFT as comparisions. Among these trackers, AutoTrack, TADT, MCCT, CCOT, ECO and DeepSTRCF are correlation filter based trackers, which has a lightweight structure and less parameters than deep learning based trackers, and the model can be deployed on limited source device. Compared with UAV 123 benchmark, challenge in UAV123@10fps dataset are more abrupt and severe. The experimental results are listed in Table 2. Compared with the correlation filter based trackers, the deep trackers, HiFT and SiamRPN++ achieve higher precision and success scores, the performance of SiamFC is closer to these correlation based trackers, SiamFC utilize the AlexNet as the backbone, but the model does not further enhance the feature representation. Our SiamHFFT model yields the best precision (76.5%) and success rate (59.5%), which has an advantage over HiFT by 1.1, 2.1%, demonstrating the effectiveness of the HFFT module, and superior robustness capacity compared to other prevalent trackers.

**Table 2:** Overall evaluation on UAV123@10fps.

	**SiamFC**	**AutoTrack**	**TADT**	**MCCT**	**SiamRPN++**	**DeepSTRCF**	**CCOT**	**ECO**	**HiFT**	**SiamHFFT**
Prec.	67.8	67.6	68.4	68.1	74.0	68.0	70.4	70.9	75.4	76.5
Succ.	47.2	48.1	50.7	49.2	55.5	49.9	50.2	51.9	57.4	59.6

The best performance are shown in red.

**Evaluation on VOT2020**: We also test SiamHFFT on the VOT2020 benchmark against HCAT, LightTrack (Yan et al., 2021b), ATOM and DiMP. VOT2020 consists of 60 videos with mask annotations and adopts the expected average overlap (EAO) as the metric for evaluating the performance of the trackers, which is calculated by:


(15) $$\begin{array}{l} \bar{\phi }N_{S}=\frac{1}{N_{S}}\sum_{i=1}^{N_{S}}\phi N_{S} \end{array}$$

where *N*_*S*_ denotes the length of the video sequences, ϕ*N*_*S*_ denotes the average accuracy of a video sequence whose length is *N*_*S*_. Finally, the EAO value can be obtained by calculating the average value of the video sequences of *N*_*S*_ length.

The experimental results are presented in Table 3. Our tracker achieves an EAO value of 0.231, robustness of 0.646, and accuracy of 0.459. The performance of SiamHFFT is comparable to that of the state-of-the-art models for each criterion.

**Table 3:** Evaluation on VOT2020.

	**HCAT**	**LightTrack**	**ATOM**	**DiMP**	**SiamHFFT**
EAO	0.276	0.242	0.271	0.274	0.231
Accuracy	0.455	0.422	0.462	0.457	0.459
Robustness	0.747	0.689	0.734	0.740	0.646

The best performance are shown in red.

### Speed, FLOPs and params

**Figure 7:**
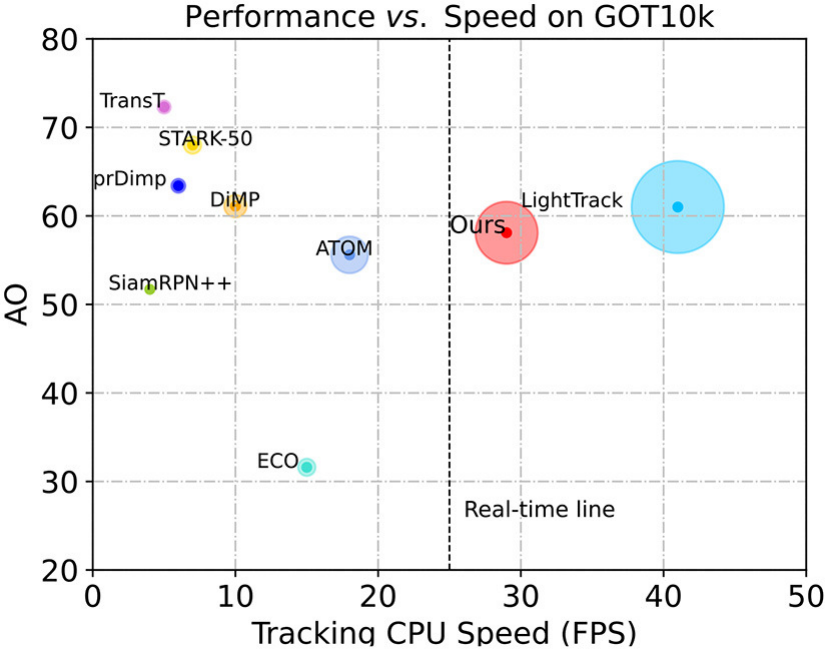
Speed and performance comparisions on GOT-10k. The horizontal axis represents model speed on a CPU and the vertical axis represents the AO score.

To verify the efficiency of our tracker, we conducted a set of experiments on the GOT-10k benchmark, which is a large-scale tracking dataset consisting of more than 10,000 videos, covering a wide range of 560 types of common moving objects. Following the test protocols of GOT-10k, all of the evaluated trackers are trained with the same training data, and are tested with the same test data. We evaluated the performance of SiamHFFT against TransT, STARK, DiMP, SiamRPN++, ECO, ATOM, and LightTrack. Our SiamHFFT is conducted on PC while the data of other trackers on GOT-10k is obtained from Chen et al. (2022b). Both average overlap (AO) and speed were considered to evaluate the performance of the trackers. We visualize the AO performance with respect to the frames-per-seconds (FPS) tracking speed. The comparision results are presented in Figure 7. Each tracker is represented by a circle, and the radius of the circle *r* is calculated as follows:


(16) $$\begin{array}{l} r\;=k\frac{speed/Average\left(speed\right)}{AO} \end{array}$$

where *k* denotes a scale factor, we set *k*=10. The higher value of *r* indicates the better performance. All trackers were tested on CPU platform, and real-time line (26 fps) performance is represented by a dotted line to measure the real-time capacity of the trackers, trackers locate on the right side of the line are considered to achieve the real-time performance. According to Figure 7, only SiamHFFT and LightTrack can meet the real-time requirement on the CPU. Among these comparision trackers, TransT utilized a modified ResNet50 as backbone and a transformer-based network to obtain discriminative features, and achieve the highest AO score, but it sacrifices the speed which runs a low speed on CPU. Similarly, STARK, DiMP, prDiMP, SiamRPN++ can only obtain satisfactory AO scores at the expense of speed. The correlation filter-based tracker, ECO, also adopts the deep features which does not achieve a satisfactory speed on CPU. Our tracker exhibits an average speed of 28 FPS on the CPU, not only reach the real-time requirement, but the area of the circle representing our method is the second large of all the trackers.

To validate the lightness of our model, we compared the floating-point operations (FLOPs) and Params of the model with STARK-S50 and SiamRPN++. FLOPs represent the theoretical calculation volume of the model, which means the number of calculations required for the inference. Params refer to the amount of the parameters in the model, which directly determines the size of the model and also directly affects the memory consumption when a model making inferences. The comparison results are illustrated in Table 4. It is worth note that our SiamHFFT tracker achieve a promising result over other trackers. The FLOPs and Parameters are 16 × and 5 × less than those of STARK-S50. This shows that our method can use fewer parameters and lower memory consumption, making it possible for deployments in the edge hardware environments.

**Table 4:** Comparision about the FLOPs and params.

**Trackers**	**FLOPs (G)**	**Params (M)**
STARK-S50	10.5	23.3
SiamRPN++	48.9	54
SiamHFFT	0.6	4.4

### Ablation studies

**Figure 8:**
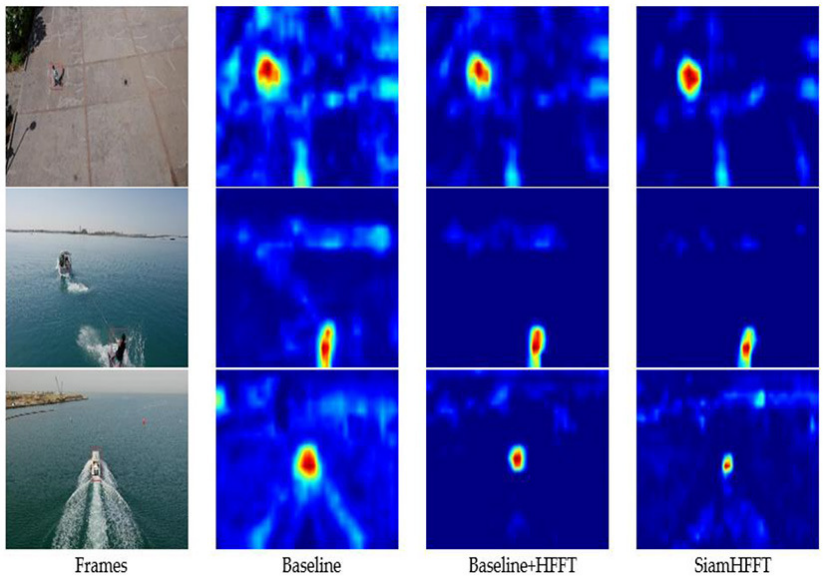
Visualization of the confidence maps of three trackers on several sequences from the UAV123 dataset. The response visualization results are an intuitive reflection of tracker performance.

This section presents ablation studies conducted to verify the effectiveness of our framework. We selected several challenging frames from the UAV123 dataset and visualized the tracking results using heatmaps, as shown in Figure 8. The first column presents the given target which is highlighted with a red box, and the remaining columns present the visualized results of the predicted target prior to the current frame.

The second column presents the visualization results of the baseline, which only adopts ShuffleNetV2 as backbone with the reshaping module and the prediction head. The response area of the baseline is much larger than the original target size and has obscure edges affected by distractors in the frames.

The third column presents the visualization results of the baseline with the HFFT module. Compared with the baseline alone, the response area is smaller and clearer because the HFFT module enhances the critical semantic and spatial features of the target, enabling the model to generate more discriminative response maps. With the HFFT module, our tracker achieves significant improvement in tracking accuracy, which validates the effectiveness of the HFFT module for handling small objects.

The last column presents the response map generated by the proposed SiamHFFT, which adopts the entire operation module, backbone, reshaping module, HFFT module and the SAM, where the classification and regression head are utilized to estimate the location of a target. According to the visualization results of the response maps, our SiamHFFT model has clear advantages over other modified versions. The response areas are more precise and discriminative relative to the distractors.

We also test the performance on UAV123 benchmark with different backbones, we use the accuracy score to measure the performance variation. Experimental result is shown in Table 5, we choose two lightweight networks, AlexNet and ShuffleNetV2, to make a comparision. Similar to Figure 8, the effectiveness of the HFFT module is measured in a quantitative manner. The model adopts ShuffleNetV2 as backbone has better performance on all of the three criteria. The experiment results of Table 4 also demonstrate the effectiveness of the HFFT module.

**Table 5:** Experimental results on UAV 123 benchmark with different backbones.

	**Baseline**	**Baseline+HFFT**	**SiamHFFT**
AlexNet	73.6	77.2	78.9
ShuffleNetV2	74.1	81.6	82.8

## Conclusion

In this paper, an HFFT tracking method based on a Siamese network was proposed. To integrate and optimize multi-level features, we designed a novel feature fusion transformer that can reinforce semantic information and spatial details during the tracking process. Additionally, based on our lightweight backbone, excessive computation for feature extraction is avoided, which accelerates object tracking speed. To validate the effectiveness of our trackers, extensive experiments were conducted on five benchmarks. Our method achieves excellent results on small target datasets such as UVA123 and UAV123@10fps, and also shows good performance on genetic public visual tracking datasets, such as LaSOT, VOT2020, and GOT-10k. Our method can potentially inspire further research on small object tracking, particularly for UAV tracking.

## Data availability statement

The original contributions presented in the study are included in the article/Supplementary material, further inquiries can be directed to the corresponding author/s.

## Author contributions

Conceptualization, methodology, software, validation, formal analysis, data curation, and writing—original draft preparation: JD. Investigation: SW. Resources, writing—review and editing, supervision, and funding acquisition: YC. Visualization: YF. All authors have read and agreed to the published version of the manuscript.

## Funding

YC is the leader of the funding for the research of National Science Foundation of China (No. 11975066), he received his Ph.D. degree from Jilin University, in 2002. He is a Professor with Jilin University, and also with Dalian University of Technology. His research interests include CMOS image sensor and digital signal processing of images.

## Conflict of interest

The authors declare that the research was conducted in the absence of any commercial or financial relationships that could be construed as a potential conflict of interest.

## Publisher's note

All claims expressed in this article are solely those of the authors and do not necessarily represent those of their affiliated organizations, or those of the publisher, the editors and the reviewers. Any product that may be evaluated in this article, or claim that may be made by its manufacturer, is not guaranteed or endorsed by the publisher.
