# Supplementary material for: Siamese hierarchical feature fusion transformer for efficient tracking
Source: Front Neurorobot. 2022 Dec 1;16:1082346. doi: 10.3389/fnbot.2022.1082346 (PMC9752039; doi:10.3389/fnbot.2022.1082346)
Supplement: Supplementary file 1 [file Data_Sheet_1.docx]

# Supplementary materials

# A. Table of abbreviations and symbols

| **Abbreviation** |  | **Symbol** |  |
| --- | --- | --- | --- |
| HFFT | Hierarchical feature fusion transformer |  | Cross-correlation operator |
| SAM | Self-attention module | $\cup$ | Union operator |
| ViT | Vision Transformer | $\cap$ | Intersection operator |
| UAV | Unmanned aerial vehicle |  | The number of pixels in the resulted region. |
| NLP | Natural language processing | Z | Template frame patch |
| NMS | Non-maximum suppression | X | Search frame patch |
| MLP | Multilayer perceptron |  | Source feature |
| GELU | Gaussian error linear unit | $\theta$ | Target feature |
| LN | Layer normalization | $b_{t}$ | Estimated bounding box |
| OPE | One pass evaluation | $b_{g}$ | Ground truth bounding box |
| CLE | Center location error | $N_{S}$ | The length of the video sequences |
| EAO | Expected average overlap | $k$ | Scale factor (set to 10) |
| FLOPs | Floating-point operations |  |  |

**B. Table of experiment parameters**

| **Train** |  | **Test** |  |
| --- | --- | --- | --- |
| Batch_size | 32 | Search_factor | 5.0 |
| epoch | 150 | Search_size | 320 |
| LR | 0.0001 | Template_factor | 1.5 |
| Weight_decay | 0.00001 | Template_size | 80 |
| Optimizer | adamw | Hanning_factor | 0.01 |
| $\lambda_{cls}$ | 2 |  |  |
| $\lambda_{giou}$ | 2 |  |  |
| $\lambda_{loc}$ | 5 |  |  |
| Search_center_jitter | 4.5 |  |  |
| Search_factor | 5.0 |  |  |
| Search_size | 320 |  |  |
| Template_center_jitter | 0 |  |  |
| Template _factor | 1.5 |  |  |
| Template _size | 80 |  |  |
